# Prevalence of Cryptorchidism Among Bulgarian Boys

**DOI:** 10.4008/jcrpe.v1i2.42

**Published:** 2008-11-03

**Authors:** Philip Kumanov, Analia Tomova, Ralitsa Robeva, Stanislav Hubaveshki

**Affiliations:** 1 Clinical Center of Endocrinology, Medical University−Sofia, Sofia, Bulgaria; +359−2 989 19 20+359−2 987 41 45phkumanov@lycos.comClinical Center of Endocrinology, Medical University−Sofia, Bulgaria 6, Dame Gruev str., 1303, Sofia, Bulgaria

**Keywords:** Cryptorchidism, prevalence, hypospadias

## Abstract

**Background**: Cryptorchidism is the most common congenital defect of the male urogenital system. It may be an important cause for male infertility. The data about its prevalence in South−eastern European countries and especially for the Balkan region are still incomplete.

**Objective**: The aim of the present study was to establish the prevalence of the abnormality in the Bulgarian population living in the different areas of the country.

**Methods**: In a population–based cross−sectional study in Bulgaria 6200 Caucasian boys (aged under 1 year to 19 years) from five regions of the country were included. One physician examined all children in order to reduce the inter−observer error.

**Results**: The prevalence of cryptorchidism was 1.52% for the total group. It was 3.2% for boys under one year of age, 2.1% for those between one and ten years and dropped significantly in older boys (0.6%). No regional or seasonal trends were established.

**Conclusions**: Our study suggests a relatively higher prevalence of cryptorchidism in Bulgaria for children younger than one year of age, while the overall prevalence is comparable to those reported for other countries. The prevalence of cryptorchidism did not differ significantly from the prevalence reported thirty years ago.

**Conflict of interest:**None declared.

## INTRODUCTION

Cryptorchidism is a condition in which one or both of the testes are not fully descended to the bottom of the scrotum. It is one of the most frequent developmental abnormalities in men and may be a prominent cause of male infertility.(1) Recently, it has been suggested that testicular cancer, poor semen quality, hypospadias and undescended testes might all be symptoms of one underlying abnormality, namely the testicular dysgenesis syndrome (TDS).(2) There is evidence to suspect that changes in lifestyle and increasing environmental exposures could be the causes underlying the upward trends in occurrence of male reproductive health problems such as gonadal cancer, undescended testis and poor semen quality.(3)

Numerous epidemiological studies indicate that the incidence of hypospadias and cryptorchidism has increased in many countries. Large regional differences have also been described. However, registry−based studies do not permit reliable comparisons and the data contain many uncertainties.(4) Consequently, prospective studies with standard inclusion criteria seem more reliable in establishing the temporal and regional trends of these congenital defects.

Thorough analyses and comparisons on the prevalence of cryptorchidism have been accomplished in and between Nordic countries and Great Britain, while published data for South−eastern European countries and especially for the Balkan region are insufficient. Therefore, we investigated the prevalence of cryptorchidism in a group of boys from different regions of Bulgaria.

## METHODS

**Figure 1:**
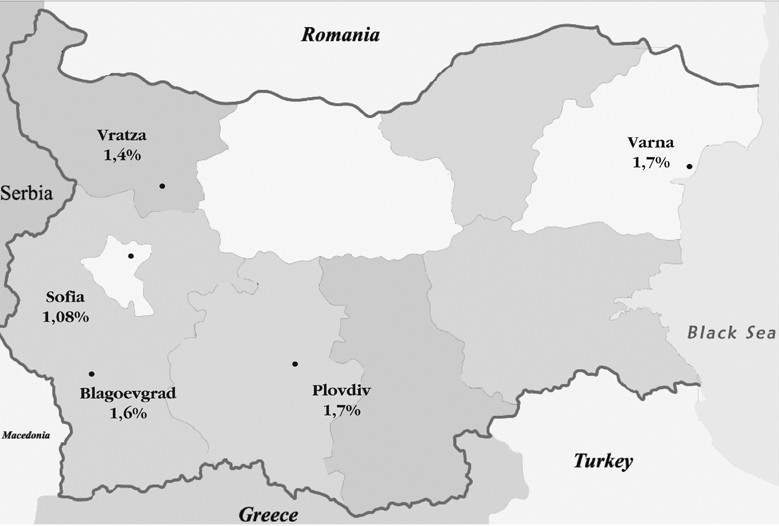
Map of Bulgaria showing prevalence of the cryptorchidism in the different regions.

Caucasian boys of ages ranging from under 1 year to 19 years (20 age groups) and residing in five regions of Bulgaria (Sofia−city, Plovdiv−city and the adjacent rural area, Varna−city and the adjacent rural area, Blagoevgrad−town and the adjacent rural area, and Vratza−town and the adjacent rural area) were included in this population−based crosssectional study (Figure 1). Sofia−city is the capital of Bulgaria with an approximate population of 1,376,000. Varna and Plovdiv are the largest Bulgarian towns with populations of about 350,000 each, while Blagoevgrad and Vratza are smaller towns with populations of around 70,000 citizens each. The total population of the country is about 8 millions. The areas and towns were chosen at random and they are representative for the country’s population and structure. Altitudes of the respective towns and villages were also recorded. Consent was obtained from all boys and/or their parents for the examination. The study was approved by the Institutional Review Board, Clinical Centre of Endocrinology, Medical University, Sofia, Bulgaria.

Every boy was assigned to a corresponding age group according to his completed age on the day of the examination. The boys were permanent residents of the respective city, town or village. There were equal numbers of boys from urban and rural areas in the regions, except for the capital. In every age group there were 310 children. The number of boys for the country and for the regions was chosen with assumed prevalence of 5% and 2% absolute precision.(5) According to this evaluation the number of all boys examined (n=6200) in the five towns and rural areas was adequate to be representative for the general population. The infants and children were examined at random in schools, kindergardens and in the Children’s Consultation (a health structure that provides medical care for all Bulgarian children). All were clinically healthy on the day of examination and the examination included palpation of the testes in all subjects.

The study was undertaken during 1990s. Only one experienced physician (P Kumanov) examined all children in order to reduce the inter−observer error. All cases of not fully descended testis were registered. Testicular position was recorded after firm and cautious traction with warm hands of the testis to the most distal position on the pathway of normal descent. Boys were diagnosed as cryptorchid if one or both testes were non−palpable, or when they could not be manipulated to a stable position in the scrotum. Retractile testes could be manipulated to a stable scrotal position and were not considered cryptorchid according to Pierik et al.(6) In the boys evaluated as cases of retentio testis, the gonad could be manipulated to the scrotum but after release of the traction it returned to abnormal position. Therefore these cases were classified as cryptorchid.(6) We did not find ectopic testis. The position of the testis was classified as follows: non−palpable, inguinal at scrotal entrance, not remaining in the scrotum after the manipulation, and remaining stable in the scrotum. The laterality of the abnormality was also recorded (left, right, or bilateral).

The children already treated for undescended testis were verified by their medical documentation records (data for orchiopexy or drug treatment), while the newly found cases were sent to hospitals, where the diagnosis was confirmed and the children received appropriate diagnostic tests and treatment.

The statistical analyses were performed using SPSS for Windows version 11.0 (Chicago, IL, USA). Frequency analyses and descriptive statistics were used, where appropriate. Differences between groups were tested with Chi−square and Fisher’s exact test. For multiple comparisons the Bonferroni test (equal variances assumed) and Tamhane’s T2 test (unequal variances assumed) were applied.

## RESULTS

We found 94 cases of cryptorchidism among all studied boys (n=6200). In 23 of them the gonad was found to be inguinal at scrotal entrance or retentio testis was established. In the other 71 boys the testis/testes could not be palpated at all. In the first group (n=23) 17 cases were right sided, four were left sided and the remaining two were bilateral. In the other group without palpable testes (n=71), 35 of cases were right sided, 30 were left sided and in six children both testes were impalpable. The differences between the prevalence of right−sided and left−sided cryptorchidism in all affected children under 1 year of age reached statistical significance (p=0.020) and tended to be significant for the whole group of all ages (p=0.052).

As expected, the prevalence of cryptorchidism among Bulgarian children was highest in the age group under one year (3.22%), while at one year of age it decreased to 2.3%. The frequency of the anomaly was 1.43% among the children between 1 and 19 years and 1.52% for all boys. The prevalence was higher in children between one and ten years (2.1%) and dropped significantly in older boys to 0.6% (p<0.001) (Figure 2). 

The prevalence of cryptorchidism in the boys of urban origin was 1.38%, and in the boys from the rural origin 1.72% (p>0.05). The regional distribution is shown on Table 1 and the age distribution is shown on the Table 2. No significant differences between the regions were found. There were also no differences in the prevalence of the cryptorchidism according to the altitudes of the places, where the children lived. The lowest prevalence of cryptorchidism was found in Sofia−city (1.08%), while in the other regions the mean prevalence was 1.62%. No seasonal differences in the prevalence of cryptorchidism according to month of birth were established. The children under one year of age were divided in four groups according to their age (1−3 months, 4−6 months, 7−9 months, 10−12 months). No differences were found in the prevalence of cryptorchidism between these groups.

**Figure 2:**
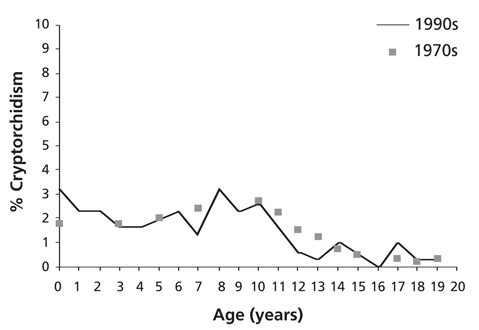
Prevalence of cryptorchidism among Bulgarian boys in the seventies (Stajkov et al.)^7^ and in the recent study.

**Table 1:**
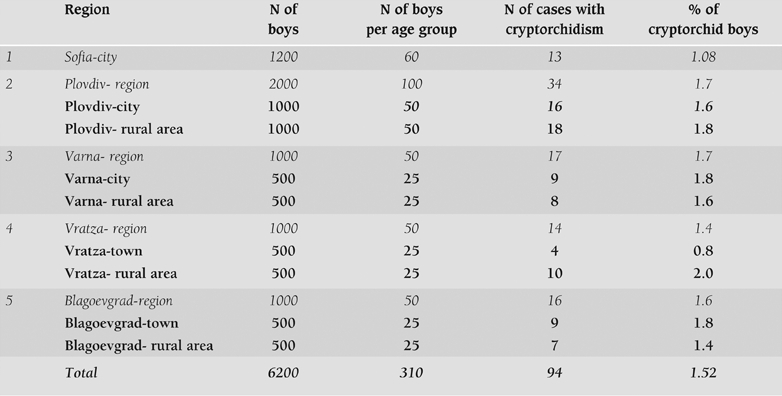
The regional distribution of the cases with cryptorchidism in Bulgaria.

**Table 2:**
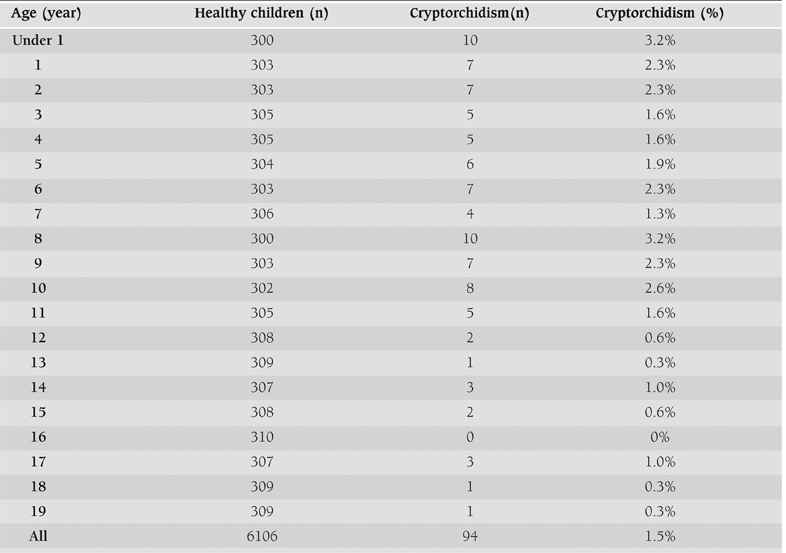
Distribution of cryptorchidism prevalence by age (completed years) groups.

## DISCUSSION

In a population–based cross−sectional study accomplished in 1990s we found that the prevalence of cryptorchidism among Bulgarian boys was 1.52%. The capital of the country and the four districts with different geographical characteristics, environmental conditions, chemical industries and altitudes, divided into rural and urban areas, were included in this study. No significant regional differences in the prevalence of the cryptorchidism were observed.

Pesticides and fertilizers used in agricultural farms are supposed to predispose to malformation as endocrine disruptors. In places closer to sea level usually occupation in agriculture predominates, in contrast to areas of higher altitude. Therefore, we compared the prevalence of cryptorchidism according to the altitudes of the villages and towns, where the study was undertaken, but no differences were found.

Our results are very similar to those in a former prospective study among 360,440 Bulgarian males (from birth to age 20 years) which was conducted about 30 years ago using the same classification system.(7) The prevalence of cryptorchidism was 1.74% (Figure 2), which is not significantly different from the prevalence of 1.52%, found in our study. The earlier study was based on reports of different paediatricians, while in our study all children were examined by one physician. Only for the age group under 1 year the present study showed approximately a two times higher frequency (3.2%) in comparison to that reported in the seventies (≍1.7%).

The regional prevalence did not differ considerably between the two studies. Prevalence for the region of Plovdiv in the 1970s was 1.36%(7), while in the 1990s the corresponding frequency was 1.7%. Interestingly, the lowest prevalence of cryptorchidism in the present study (1.08%) was found in Sofia−city. Based on these two studies, we can state that no significant increase in the prevalence of the cryptorchidism could be established for the last thirty years in Bulgaria.

Despite the differences in methodology, the established prevalence of undescended testes in our country was relatively high for younger children in comparison to other countries. For instance, the prevalence among schoolboys from 6 to 15 years of age in the neighbour Turkey was reported as only 0.9%(8), while for the same age group our results showed a figure of 1.58%. The results reported in Italy were 1.22% for the children delivered at term, and 3.13% for preterm infants.(9) At 18 months after birth the prevalence in Finland was estimated to be 1% (0.5−1.6%), while in Lithuania it was 1.4% and in Denmark 1.5% (0.8− 2.5%).(10, 11) The latter data were closest to our figures (the prevalence is 2.3% among boys at age of 1 year). The sudden drop in the prevalence of the cryptorchidism in pubertal ages could be related to the spontaneous descend of the testes.

In our study the prevalence of unilateral undescended testis was greater than that of bilateral and the right sided cryptorchidism predominated over the left sided. Similarly, in the study of Pierik et al.(6) the prevalence of the unilateral cryptorchidism was 83%, while that of the bilateral were only 17%.(6) In the UK study, of the 7400 boys 1.92% had bilateral and 3.0% unilateral cryptorchidism at birth, and in the latter group 52.1% had left sided and 47.9% right sided cryptorchidism.(12) We have no explanation for these differences in the laterality of cryptorchidism.

As evident from different studies, the incidence is much higher in premature infants than in full−term babies. Since the testes normally descend during late pregnancy, undescended testes are often seen in premature children, and a spontaneous descend can be expected.(13) Sijstermans et al.(14) thoroughly analysed 46 articles to establish the undescended testis (UDT) rate. They found that in premature and/or birth weight <2.5 kg infants an UDT rate at birth varied from 1.1 to 45.3% and at 1 year from 1.9 to 7.3%, while the corresponding rates in term and/or birth weight >2.5 kg infants varied only from 1.0 to 4.6% at birth and from 1.0 to 1.5% at 1 year, respectively. Thus, one limitation of our study was that we did not specify the term and preterm boys with cryptorchidism. We did not have complete information about the birth weight of the children and its influence on the presence of cryptorchidism was not investigated.

In recent years a hypothesis for TDS was introduced. TDS involves congenital masculinisation disorders (cryptorchidism and hypospadias) and disorders in young adult men (oligozoospermia and testis cancer). The possible role of different endocrine disruptors on the maldevelopment of the fetal testis has been discussed, even though there is no definitive evidence that exposure of humans to environmental chemicals can induce testicular dysgenesis.(15, 16) Therefore, we decided to observe the components of the TDS among Bulgarian children. In the present large epidemiological study, in which the examinations were performed by one and the same investigator, the prevalence of cryptorchidism was 1.52%, while that of hypospadias was only 0.29%.(17) The prevalence of the cryptorchidism showed no seasonal or regional trends. On the contrary, the prevalence of the hypospadias tended to be higher in Bulgarian boys of rural origin. It was lower at higher altitudes (above 500 m), showed regional differences and a striking seasonal variation.(17) It could be suggested that unknown specific environmental factors may influence the hypospadias rate,but not the cryptorchidism incidence.

The relatively higher prevalence of cryptorchidism in Bulgaria for younger age groups in comparison to the neighboring countries could be due to genetic factors, as different studies have demonstrated the familial clustering of the defect.(18, 19) Numerous animal models suggested candidate genes for cryptorchidism and allow better comprehension of the mechanisms regulating testicular descent.(20) In humans, a crucial step in the last years was the discovery of the insulin like factor 3 (ILF3) as a hormonal regulator of the gubernacular differentiation, the identification of its specific receptor RXFP2 and mutation analyses of the ILF3/RXFP2 genes.(20) The influence of androgen and estrogen receptor genes polymorphisms, Y−chromosome microdeletions, as well as the role of some chromosomal alterations has also been examined in order to find the genetic causes for cryptorchidism. Unfortunately, we did not have the possibility to conduct genetic investigations on the children in this study.

Other important possibilities are the acquired forms of cryptorchidism such as ascensus of the testis, entrapment of the testis in an inguinal scar following previous hernia operation. Further studies are needed to explain the regional features and the role of the environmental and genetic factors in the development of cryptorchidism.

**Figure 2:**
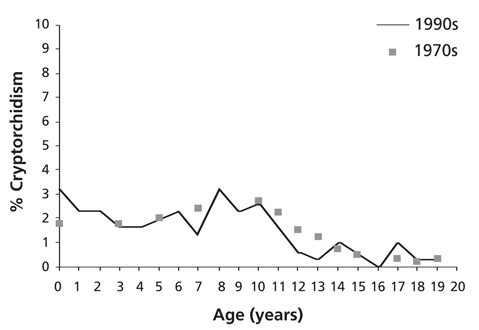
Prevalence of cryptorchidism among Bulgarian boys in the seventies (Stajkov et al.)^7^ and in the recent study.

## ACKNOWLEDGEMENT

We thank to Prof. Mircho Vukov for the statistical evaluation of the data. We express our gratitude to Katharina M. Main MD, PhD for the valuable advices and suggestions during manuscript preparation.

## References

[1] Lee PA, Kogan BA, Coughlin MT, Winters S (2004). Cryptorchidism. Male hypogonadism.

[2] Skakkebaek NE, Rajpert−De Meyts E, Main KM (2001). Testicular dysgenesis syndrome: an increasingly common developmental disorder with environmental aspects: Opinion.. Hum Reprod.

[3] Skakkebaek NE, Jørgensen N, Main KM, Rajpert−De Meyts E, Leffers H, Andersson A−M, Juul A, Carlsen E, Mortensen GK, Jensen TK, Toppari J (2006). Is human fecundity declining?. Int J Androl.

[4] Toppari J, Kaleva M, Virtanen HE (2001). Trends in the incidence of cryptorchidism and hypospadias and methodological limitations of registry−based data.. Hum Reprod Update.

[5] Lwanga SK, Lemeshow S (1991). Sample size determination in health studies : a practical manual..

[6] Pierik FH, Burdorf A, de Muinck Keizer−Schrama SM, Wolffenbuttel KP, Nijman JM, Juttmann RE, Weber RF (2005). The cryptorchidism prevalence among infants in the general population of Rotterdam, the Netherlands.. Int J Androl.

[7] Stajkov T (1982). Prevalence, characteristics and prognosis of the endocrine disorders in the People’s Republic of Bulgaria and an organization of the fight against them..

[8] Yücesan S, Dindar H, Olcay I, Okur H, Kiliçaslan S, Ergören Y, Tüysüz C, Koca M, Civilo B, Sen I (1993). Prevalence of congenital abnormalities in Turkish school children.. Eur J Epidemiol.

[9] Ghirri P, Ciulli C, Vuerich M, Cuttano A, Faraoni M, Guerrini L, Spinelli C, Tognetti S, Boldrini A (2002). Incidence at birth and natural history of cryptorchidism: a study of 10,730 consecutive male infants.. J Endocrinol Invest.

[10] Preiksa RT, Zilaitiene B, Matulevisius V, Skakkebaek NE, Petersen JH, Jørgensen N, Toppari J (2005). Higher than expected prevalence of congenital cryptorchidism in Lithuania: a study of 1204 boys at birth and 1 year follow−up.. Hum Reprod.

[11] Boisen KA, Kaleva M, Main KM, Virtanen HE, Haavisto A−M, Schmidt IM, Chellakooty M, Damgaard IN, Mau C, Reunanen M, Skakkebaek NE, Toppari J (2004). Difference in prevalence of congenital cryptorchidism in infants between two Nordic countries.. Lancet.

[12] John Radcliffe Hospital Cryptorchidism Study Group (1992). Cryptorchidism: a prospective study of 7500 consecutive male births, 1984−1988.. Arch Dis Child.

[13] Toppari J, Kaleva M (1999). Maldescendus testis.. Horm Res.

[14] Sijstermans K, Hack WWM, Meijer RW, van der Voort−Doedens LM (2008). The frequency of undescended testis from birth to adulthood: a review.. Int J Androl.

[15] Sharpe RM, Skakkebaek NE (1993). Are oestrogens involved in falling sperm counts and disorders of the male reproductive tract?. Lancet.

[16] Sharpe RM (2006). Pathways of endocrine disruption during male sexual differentiation and masculinisation.. Best Pract Res Clin Endocrinol Metab.

[17] Kumanov Ph, Robeva R, Tomova A, Hubaveshki S (2007). Prevalence of the hypospadias among Bulgarian boys − a prospective study.. Eur J Pediatr.

[18] Weidner IS, Moller H, Jensen TK, Skakkebaek NE (1999). Risk factors for cryptorchidism and hypospadias.. J Urol.

[19] Berkowitz GS, Lapinski RH (1996). Risk factors for cryptorchidism: a nested case−control study.. Paed Perinatal Epidemiol.

[20] Foresta C, Zuccarello D, Garolla A, Ferlin A (2008). Role of hormones, genes, and environment in human cryptorchidism.. Endocr Rev.

